# Synergistic Degradation of Pyrethroids by the Quorum Sensing-Regulated Carboxylesterase of *Bacillus subtilis* BSF01

**DOI:** 10.3389/fbioe.2020.00889

**Published:** 2020-07-29

**Authors:** Ying Xiao, Qiqi Lu, Xin Yi, Guohua Zhong, Jie Liu

**Affiliations:** ^1^Key Laboratory of Integrated Pest Management of Crop in South China, Ministry of Agriculture, South China Agricultural University, Guangzhou, China; ^2^Key Laboratory of Natural Pesticide and Chemical Biology, Ministry of Education, South China Agricultural University, Guangzhou, China; ^3^Guangdong Research Institute of Petrochemical and Fine Chemical Engineering, Guangzhou, China; ^4^Guangdong Laboratory for Lingnan Modern Agricultural Science and Technology, Guangzhou, China

**Keywords:** biodegradation, quorum sensing, pyrethroid, *comA*, carboxylesterase

## Abstract

The well-studied quorum sensing (QS) mechanism has established a complex knowledge system of how microorganisms behave collectively in natural ecosystems, which contributes to bridging the gap between the ecological functions of microbial communities and the molecular mechanisms of cell-to-cell communication. In particular, the ability of agrochemical degradation has been one most attractive potential of functional bacteria, but the interaction and mutual effects of intracellular degradation and intraspecific behavior remained unclear. In this study, we establish a connection between QS regulation and biodegradation by harnessing the previously isolated *Bacillus subtilis* BSF01 as a template which degrades various pyrethroids. First, we characterize the genetic and transcriptional basis of *comA*-involved QS system in *B. subtilis* BSF01 since the ComQXPA circuit coordinates group behaviors in *B. subtilis* isolates. Second, the genetic and transcriptional details of pyrethroid-degrading carboxylesterase CesB are defined, and its catalytic capacity is evaluated under different conditions. More importantly, we adopt DNA pull-down and yeast one-hybrid techniques to reveal that the enzymatic degradation of pyrethroids is initiated through QS signal regulator ComA binding to carboxylesterase gene *cesB*, highlighting the synergistic effect of QS regulation and pyrethroid degradation in *B. subtilis* BSF01. Taken together, the elucidated mechanism provides novel details on the intercellular response of functional bacteria against xenobiotic exposure, which opens up possibilities to facilitate the *in-situ* contaminant bioremediation via combining the QS-mediated strategies.

## Introduction

Energy transfer and element cycling throughout the whole eco-chain largely benefit from the ubiquitous existence of microorganisms in diverse ecosystems (Delgado-Baquerizo et al., 2016; Maron et al., 2018; Tanentzap et al., 2019). Indeed, microbes enable the persistent transformation of inorganic elements into widely available nutrient and the effective decomposition of metabolic wastes from higher organisms. In fact, scientists have proved that microbes are highly gregarious, and they have evolved a unique strategy called quorum sensing (QS) to realize cell-to-cell communication, further coordinating collective behaviors in order to fit in complex environments (Hmelo, 2017; Whiteley et al., 2017; Wang et al., 2020). After decades of effort, QS regulation has been well understood as a mechanism of bacterial intercellular communication depending on their self-produced extracellular signal molecules, which can accumulate to certain threshold levels that trigger transcription of specific genes and coordinate biological behaviors within bacterial community to survive against stresses, reproduce and function as a key ecological mediator (Even-Tov et al., 2016; Hmelo, 2017). Taking advantage of in-depth studies of QS rules, more insights and solutions are inspired to improve those bacteria-centered environmental applications, for instance, anti-biofouling and wastewater treatment (Lee et al., 2016; Kalia et al., 2019; Yu et al., 2019; Zhang et al., 2019). However, a knowledge gap is still between the molecular mechanism of QS regulation and their biological functions, requesting continuous effort to elucidate the roles of QS in natural ecosystems.

The QS system in Gram-positive *Bacillus subtilis* marked with the typical ComQXPA circuit is one of the well-studied models underlying bacterial communication (Bareia et al., 2018). Briefly, the ComX signal peptide is synthesized as autoinducer, secreted and transported by ComQ. With increasing cell density and accumulation of ComX, the extracellular ComX binds to its receptor ComP, which subsequently phosphorylates and activates ComA. The activated ComA∼P stimulates the secretion of surfactin and indirectly control production of other public good (Boguslawski et al., 2015; Kalamara et al., 2018). As the response regulator of QS signals, the role of ComA in this circuit is vital due to its direct mediation of the expression of a series of downstream genes (Wolf et al., 2015). Accordingly, the unveiled QS mechanism may lead us to a more macroscopic question: are the biological functions of *B. subtilis* related to QS regulation? And how? Another noteworthy fact about *B. subtilis* is that these spore-forming isolates are versatile in biotechnology engineering sections, and most of them are capable of being food preservatives, therapeutic agents and biopesticides (Ÿztürk et al., 2016; Liu et al., 2017; Caulier et al., 2019). Moreover, they exhibit remarkable potential in xenobiotic degradation due to their abundant intracellular enzymes, which encourages constant exploitation of *B. subtilis* for green, economical and effective approaches in environmental bioremediation (Ding et al., 2015; Tao et al., 2017; Al-Gheethi et al., 2019; Oh et al., 2019). It is widely accepted that, in response to xenobiotic pressure, the group of *B. subtilis* could harness toxic compounds as carbon/nitrogen source for nutrient metabolism, which is defined as biodegradation in the aspect of ecological functions. As mentioned before, microbial functions are regulated by QS system, but the mechanism of how *B. subtilis* responds after QS system being triggered remains unclear. In other words, it is ambiguous whether the intracellular enzyme-driven biodegradation is linked to interspecific communication of *B. subtilis* in the presence of toxic compounds. Hence, it is of great interest to reveal the role of QS system in coordinating biodegradation function in *B. subtilis*, which may help to understand bacterial ecological functions in term of their social activity and ultimately improve the *B. subtilis*-involved bioremediation of contaminated environment.

Pyrethroids are highly efficient and broad-spectrum insecticides developed rapidly since the 1970s, occupying 38% of the world insecticide market currently. As an unpleasant consequence, the excessive use of pyrethroids in agriculture and homes has made non-target organisms and even human suffered from uncertain health risks and survival threats (Li et al., 2016). *B. subtilis* is proved to degrade pyrethroids owing to its highly catalytic carboxylesterases (Bhatt et al., 2020), and previously a bacterial strain *B. subtilis* BSF01 was isolated with excellent pyrethroid-degrading ability (Xiao et al., 2015). In this study, we would establish the correlation between QS regulation and biodegradation function based on the hypothesis that QS system in *B. subtilis* BSF01 could regulate pyrethroid biodegradation by manipulating the pyrethroid-hydrolyzing carboxylesterase due to following facts: (1) carboxylesterase plays a key role in the biodegradation of pyrethroids by *B. subtilis* (Zhan et al., 2020); (2) the regulation of various catalytic processes (such as biosynthesis) has been proved to be highly related to QS system in other studies (Özcengiz and Öğülür, 2015; Wu et al., 2019); and (3) in the QS system of *B. subtilis*, ComA mediates the expression of a series of downstream genes to coordinate group behaviors. First, the genetic basis, transcriptional characters and activities were investigated within ComQXPA and CesB. Moreover, we evidenced that enzymatic degradation of pyrethroids was initiated by the binding of ComA to carboxylesterase gene *cesB*. The elucidated mechanism provides novel mechanistic details on bacterial intercellular interaction in response to pyrethroid exposure, and inspires thought-provoking platforms based on QS coordination to further utilize and improve of *in situ* contaminant bioremediation.

## Materials and Methods

### Chemicals and Chromatographic Analysis

Pyrethroids including β-cypermethrin (95%), cypermethrin (92.9%), β-cyfluthrin (95%), cyfluthrin (95.1%), *λ*-cyhalothrin (98.4%), and cyhalothrin (95%) were purchased from Dr. Ehrenstorfer GmbH (Augsburg, Germany). Each stock solution (1 g L^–1^) was prepared in acetone and stored at 4°C prior to use. The mobile solvents for high-performance liquid chromatography (HPLC) were purchased from Thermo Fisher Scientific, Inc. (Fair Lawn, NJ, United States). Ampicillin (AMP), Kanamycin (Kan) and isopropyl-β-D-thiogalactopyranoside (IPTG) were purchased from Merck (Germany), Aureobasidin A (AbA) was purchased from Clontech (United States). Other chemicals and solvents involved in this study were of analytical grade.

To calibrate the standard curves, the working solutions of each pyrethroid were prepared by dilution the stock solution into a series of concentrations (at 0.1, 0.5, 1, 2, and 5 μg mL^–1^) to determine the retention time and the linear regressions by chromatographic analyses. An Agilent 1260 HPLC system was used with an Agilent ZORBAX SB-C_18_ reversed phase column (4.6 nm × 150 mm, 5 μm) at 235 nm wavelengths. The mobile phase comprising acetonitrile and water (85:15, *v*/*v*) was used at the flow rate of 1.0 mL/min and 10 μL of injection. More details are described in Supplementary Material including the calibration of standards and the spiked recoveries of six applied pyrethroids (Supplementary Table S1).

### Strains, Plasmids, and Medium

The strains, plasmids and medium used in this study are listed in Table 1. *B. subtilis* BSF01 was isolated previously, showing pyrethroid-degrading capacity (Xiao et al., 2015). *Escherichia coli* DH5α and plasmid pMD19-T were used for cloning the QS transcription regulator gene *comA* and carboxylesterase gene *cesB* in *B. subtilis* BSF01. *E*. *coli* BL21 (DE3) was used as the host for recombinant expression of ComA and CesB, which were constructed in the plasmids pET-32a (+) and pET-SUMO (Novagen, United States), respectively. For yeast one-hybrid (Y1H) assay, pAbAi and pGADT7 were used as bait plasmid and prey plasmid, respectively, which were all used *E*. *coli* TOP 10 as host to grow. The mineral salt medium (MSM) and Luria-Bertani medium (LBM) were used for growing B. subtilis BSF01 and other strains involved at 32 and 37°C, respectively. For the *E. coli* transformants, AMP or Kan was added to avoid infection.

**TABLE 1:** Strains, plasmids, and medium used in this study.

Strains, plasmids, and medium	Description	Source/reference
**Strains**		
*B. subtilis* BSF01	Wild-type, isolated from activated sludge samples	Xiao et al., 2015
*Escherichia coli (E. coli)* DH5α	F-, φ80d*lac*ZΔM15, Δ(lacZY-argF)U169, *deoR, recA*1, *endA*1, *hsdR*17(rK^–^mK^+^), *phoA, sup*E44, λ^–^, *thi*-1 *gyr*A96, *relA1*.	TaKaRa, Japan
*E. coli* BL21 (DE3)	F-*omp*T, hsdS(rB–mB–) galλ(DE3)	TaKaRa, Japan
*E. coli* TOP 10	F-, mcrAΔ(mrr-hsd RMS-mcrBC), Φ80, *lac*ZΔM15, Δ*lac*X74, *recA*1, *ara*Δ139Δ(ara-leu)7697, galU/galK, rps, (StrR) endA1, nupG.	TaKaRa, Japan
Y1HGold	*MAT*α, *ura*3-52, *his*3-200, *ade*2-101, *trp*1-901, *leu*2-3, 112, *gal*4Δ, *gal*80Δ, *met*–, *MEL*1	Clontech, United States
**Plasmids**		
pMD19-T	*E. coli* cloning vector; Amp^r^	TaKaRa, Japan
pET-32a (+)	*E. coli* expression vector with 6× His-tag at C-terminus and center, and a thioredoxin-tag at N-terminus, T7 RNA polymerase gene promoter and terminator; Amp^r^	Novagen, United States
pET-SUMO	*E. coli* expression vector with a 6× His-tag and a 109-amino acid SUMO (Small Ubiquitin-Related Modifier) solubility tag at the N-terminus, T7 RNA polymerase gene promoter and terminator; Kan^r^	Novagen, United States
pAbAi	Yeast reporter vector used in one-hybrid assays, contains yeast iso-1-cytochrome C minimal promoter and the AUR1-C gene, AbA^r^	Clontech, United States
PGADT7	Yeast expression vector; Amp^r^	Clontech, United States
pMD19-*comA*	pMD19-T simple derivate, containing the *comA* gene from *B. subtilis* BSF01	This study
pMD19-*CesB*	pMD19-T simple derivate, containing the *CesB* gene from *B. subtilis* BSF01	
pET-32a-*CesB*	7.8 kb pET-32a derivate carrying the *CesB* gene	
pET-SUMO-*comA*	7.3 kb pET-32a derivate carrying the *comA* gene	
pAbAi-*CesB*	6.8 kb pAbAi reporter carrying the *CesB* gene	
pGADT7-*comA*	8.6 kb pGADT7 -prey vector carrying the *comA* gene	
**Medium**		
LB	Yeast extract 0.5%, Peptone 1.0%, Sodium chloride 1.0%, Agar powder 1.5% (solids), pH 7.0	
MSM	(NH4)_2_SO_4_ 2.0 g, MgSO_4_⋅7H_2_O 0.2 g, CaCl_2_⋅2H_2_O 0.01 g, FeSO_4_⋅7H_2_O 0.001 g, Na_2_HPO_4_⋅12H_2_O 1.5 g, KH_2_PO_4_ 1.5 g, pH 7.0	
YPDA	Yeast extract 1.0%, Peptone 2.0%, Glycerol 2.0%, Ade 0.3%, Agar 1.5%, pH 6.5	

### Cloning and Construction of Expression Vector of the Transcription Regulator Gene *comA* in BSF01

Genomic DNA was extracted from BSF01 using MiniBEST Bacteria Genomic DNA Extraction Kit Ver.3.0 (TaKaRa, Japan) according to the manufacturer’s protocol. Based on the type culture *B. subtilis* 168 (NC 000964.3) genome information from National Center for Biotechnology Information (NCBI), an open reading frame (ORF) sequence of *comA* gene (NP 391046.1, 645 bp) was PCR amplified with the specific primers of *comA*-F and *comA*-R (Supplementary Material). The PCR was performed using the following program: 95°C for 3 min, followed by 30 cycles (95°C for 3 s, 54°C for 30 s, and 72°C for 1 min), and a final extension at 72°C for 10 min. After detecting with 1.2% agarose gel electrophoresis, the PCR products were purified and ligated into the pMD19-T Simple vector and transformed into *E. coli* DH5α cells. Then the selection of transforms was performed on LB agar plates containing AMP (100 mg L^–1^) and sequenced by Thermo Fisher Scientific (United States) after purification or IGE Biotechnology LTD (Guangzhou, China) after purification to obtain the positive recombinant colony. To express ComA protein, the complete sequence of *comA* gene was cloned with the primers of *comA*-eF and *comA*-eR. After the PCR products were purified and ligated into the pMD19-T Simple vector and sequenced, the positive recombinant plasmids were digested with *Xhol* I and *Eco R* I, and the gene was inserted into the pET-SUMO expression plasmid to generate the pET-SUMO-ComA plasmid. And the correct recombinant plasmid was transformed into competent *E. coli* BL21 (DE3) for protein expression.

### Expression and Purification of ComA Protein

The expression of ComA protein was carried out as reported with slight modification (Zhou et al., 2018). Briefly, the *E. coli* BL21 (DE3) harboring the pET-SUMO-*comA* plasmid were cultured in 1 mL of LB broth with Kan (100 μg L^–1^) at 200 rpm and 37°C overnight. The overnight culture was transferred into the fresh LB medium by 1% of the inoculation. After 4 h of incubation, the protein expression was induced with a final concentration of 0.5 mM IPTG, and culturing continued at 150 rpm and 16°C overnight. The bacterial cells were harvested by centrifugation at 7000 rpm and 4°C for 15 min, re-suspended in phosphate buffer solution (PBS, pH 7.5) and disrupted by ultrasonication for 30 cycles (treated 4 s and then halted 8 s) in ice bath. The cell debris was removed by centrifugation at 12,000 rpm and 4°C for 20 min. Then, the supernatant was collected and purified with WorkBeads 40 Ni (Tiangen Biotech, Beijing, China) according to the manufacturer’s recommendations. The purified ComA was quantified by using Bradford Protein Assay Kit (Tiangen Biotech, Beijing, China) according to the manufacturer’s recommendations and analyzed by SDS-PAGE (15% gels). After electrophoresis, the gels were stained with 0.1% Coomassie brilliant blue R-250 to analyze the molecular weight of ComA.

### Cloning and Expression and Purification of Carboxylesterase CesB in *B. subtilis* BSF01

For cloning the ORF sequence of carboxylesterase gene *cesb*, we designed the specific oligonucleotide primers using the Primer Premier 5.0 software as referred to the *cesb* sequence (NP 388108.1, 891 bp) of *B. subtilis* 168 in NCBI (Supplementary Material). The PCR Genomic DNA of strain BSF01 was as template and the ORF sequence of *cesb* was cloned by using the same methods described above. For expression of carboxylesterase CesB, the complete sequence of *cesB* gene was amplified by applying primers of *cesB*-eF and *cesB*-eR (Supplementary Material). And the PCR products was digested by *BamH* I and *Hind* III, and inserted into expression vector pET-32a (+) with a C-terminal 6×-His-tag, which had also been digested with the same restriction enzymes as *cesB* products to generate the pET32a (+)-*cesB* plasmid. Then the recombinant plasmid was transformed into competent *E. coli* BL21 (DE3) and plated on LB agar plates with 100 μg mL^–1^ of Amp to select the positive recombinant colony. After sequencing, the expression and purification of CesB protein were carried out as section “Expression and Purification of ComA Protein.”

### Sequence Analysis of ComA and CesB Based on Bioinformatics

The correctly amplified sequence of *comA* and *cesB* were processed and translated by DNASTAR Lasergene software for bioinformatics analysis. The DNA and protein sequence alignments were performed at NCBI using the BLASTN and BLASTP programs^1^, respectively. Molecular mass and pI calculations of protein ComA and CesB were predicted using the ExPASy ProtParam Server^2^. Protein secondary structure was predicted by using NPS@ web server^3^. Multiple amino acid sequences alignment was based on Clustal W 2.0. The conserved domains were identified via the NCBI website^4^.

### Molecular Dynamics Simulations and Site-Directed Mutagenesis

All molecular dynamics simulations were performed with Discovery Studio 4.5. After amino acid sequences multiple alignments, chain B of carboxylesterase CesB from *B. subtilis* (Protein Data Bank accession code 4CCY) was served as the template for homology modeling. Profile-3D program and Ramachandran plot were used for rational evaluation of the 3D model, which was energy minimized by the CHARMm forcefield (Godinho et al., 2012). Form Receptor Cavities and CDOCKER programs were applied for the construction of ligand β-cypermethrin. Molecular docking of β-cypermethrin and the 4CCY was performed with approach CDOCKER. All structures were further energy minimized using CHARMm forcefield. The optimal docking complex was selected by the Consensus Score program.

In order to obtain the key amino acids of docking complex, Calculate Mutation Energy was adopted to conduct Computational alanine scanning mutagenesis (ASM) among the amino acid residues that were located near the ligand β-cypermethrin of docking complex within the distance of 4 Å. The corresponding amino acid residues that were with less affinity and larger total energy were screened for further saturated mutagenesis. Five key amino acid residues in the docking complex were selected according to the results of the saturation mutagenesis. With the aid of Primer X website^5^, five pairs of primers were designed and listed in Supplementary Material. Site-directed mutagenesis assays were performed with Fast Site-Directed Mutagenesis Kit (Tiangen Biotech, Beijing, China) following to the producer’s protocol.

### Enzyme Assay

The enzymatic capacities against various pyrethroids were conducted in PBS (pH 7.5) at 37°C in water bath for 2 h as a default setting. Basically, the reactions were performed in sterilized 1.5 mL centrifuge tubes containing 0.5 mL PBS, 10 μL purified carboxylesterase CesB and defined volumes of pyrethroids to obtain a final concentration of 20 mg L^–1^. The mixtures were incubated for 2 h and then supplemented with 0.5 mL acetonitrile for ultrasonic extraction. Suitable amount of sodium chloride was added to make the solutions saturated. The supernatant phase was used to determine the pyrethroid residues by HPLC. One activity unit (U) was defined as the amount of enzyme needed to catalyze 1 μg of the pyrethroids per minute. The optimal reaction conditions and substrate of the enzyme activity was expressed as 100%, and the others were represented as relative enzyme activity.

For the substrate specificity assay, six pyrethroids including β-cypermethrin, cypermethrin, β-cyfluthrin, cyfluthrin, λ-cyhalothrin and cyhalothrin were applied. All reaction occurred at pH 7.5 and 37°C with the initial concentration of 20 mg L^–1^. Subsequently, the optimal reaction temperature and pH were determined by applying 20 mg L^–1^ β-cypermethrin with various pH values (pH 5.0–9.0) at 37°C, or under different temperatures ranging from 20 to 50°C, pH 7.5, followed by measuring the degradation activity under water bath for 1 h. And no enzyme-added group was used as control.

### qRT-PCR for Relative Expressions of Gene *cesB* and *comA*

Total RNA of *B. subtilis* BSF01 was extracted by Eastep^TM^ Total RNA Extraction Kit (Promega, United States), which was served as the template for reverse transcription using FastQuant RT Kit (with gDNase) (Tiangen Biotech, Beijing, China) according to the manufacturer’s instructions. Gene-specific primers used for *cesB* and *ComA* transcription assessment were designed using the Primer 5.0 software and listed in Supplementary Material. qPCR reactions were carried out on CFX Connect Real-Time PCR Detection System (Bio-Rad, United States) with iTaq^TM^ Universal SYBR^®^ Green Supermix (Bio-Rad, United States). The transcriptional levels of *cesB* and *comA* were normalized with respect to the 16S *rRNA* expression that was used as an internal reference (Yong et al., 2011). No β-cypermethrin was given to the control reactions. The thermal cycling program was as follows: pre-denaturation at 95°C for 30 s, then 40 cycles of denaturation at 95°C for 10 s, annealing at 60°C for 10 s, and extension at 72°C for 30 s. The relative gene expression levels were calculated according to the threshold cycle method (2^–ΔΔCT^) as described (Livak and Schmittgen, 2001). The qPCR analysis was performed with three biological and three technical replicates, and the results from experimental replicates were expressed as the mean (±SEM) and analyzed by using SPSS 17.0 (SPSS Inc., Chicago, IL, United States).

### The Binding of Gene *cesB* and Transcription Regulator ComA by DNA Pull-Down

The DNA pull-down assay was conducted as reported with modification (Xie et al., 2016). Briefly, the promoter probe of gene *CesB* was obtained by amplification using synthetic specific biotinylated primers (Thermo Fisher Scientific, United States), which were shown in Supplementary Material. We premixed 5 μg of biotinylated DNA (promoter probe) with 500 μg of ComA protein on ice, and the mixture was incubated with 100 μL of streptavidin-agaroseG beads for 1 h at 4°C. Following incubation, owing to the affinity of the streptavidin-agarose beads with biotinylated DNA, the DNA-protein complex was pulled down by centrifugation at 5000 rpm for 30 s at 4°C to remove the supernatant. Then, the beads were washed three times with ice-cold PBS buffer. After the last wash, the pull-down mixture was re-suspended in distilled water at 70°C for 3 min to break the bond between streptavidin and biotin. The eluted proteins from the beads was analyzed by SDS-PAGE and silver staining observation (Tran et al., 2015). No ComA protein was added to the negative control.

### Verification of Binding by Y1H Assay

To verify the results from DNA pull-down assay, the Y1H assay was performed using Yeastmaker^TM^ Gold Yeast One-Hybrid Library Screening System (Clontech, United States). The promotor region (bait) of *cesB* was amplified and inserted into the vector pAbAi to construct reporter plasmid, pAbAi-*cesB*. The coding fragments of *comA* was amplified using thermostable DNA polymerase from Thermococcus kodakarensis (KOD) polymerase (Toyobo) and was sub-cloned into pGADT7 vector to construct the prey plasmid, pGADT7-*comA*. After restriction digestion, the linear pAbAi-bait was transformed into the yeast strain Y1HGold to generate Y1HGold-pAbAi-bait. Followed by introducing the pGADT7-prey vector into the recombinant strain Y1HGold-pAbAi-bait, the transformed yeast cells were plated onto SD/-Leu/AbA medium with AbA of 200 ng mL^–1^ for stringent screening of the possible interactions. If the encoded protein ComA possessed the ability of activating *cesB*, it would motivate the expression of the reporter gene, leading to the growth of the yeast cells on the medium (Sun et al., 2017). Besides, the recombinant strain carrying pGADT7 and pGADT7-p53 were used for the negative and positive controls, respectively. All primers used for cloning these constructs are listed in Supplementary Material.

### Nucleotide Sequence Accession Numbers

GenBank accession numbers were assigned for the transcription regulator gene *comA* sequence (MG604348) and the carboxylesterase gene *cesB* sequence (MG604349) of *B. subtilis* strain BSF01.

## Results and Discussion

### *comA*-Coordinated QS System in *B. subtilis* BSF01

**FIGURE 1:**
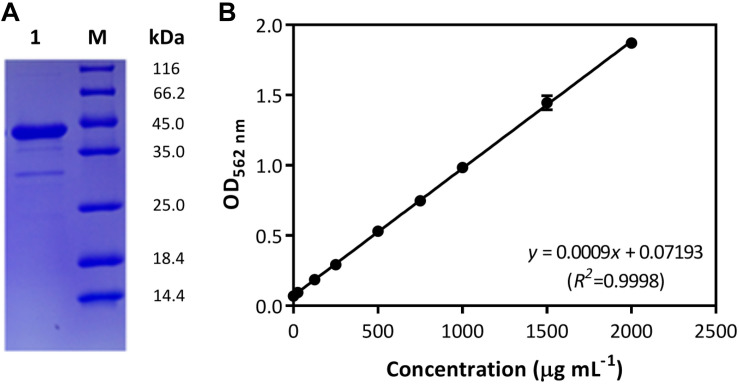
Characterization of ComA protein. **(A)** The purification of the recombinant pET-SUMO-*comA* protein (M, protein marker; lane 1, purified recombinant); **(B)** the calibration of BSA quantification (*y* = 0.0009*x* + 0.07193, *R*^2^ = 00 where *x* is the BSA concentration and *y* is related optical absorption at OD_562 nm_).

In the Gram-positive bacterium *B. subtilis*, the *comA*-involved QS regulation for cellular cooperative behaviors has been well studied, in which the role of ComA is crucial for cytoplasmic control of the production of surfactin and other public goods (Wolf et al., 2015; Kalamara et al., 2018). Due to the polymorphy of encoding systems among *B. subtilis* isolates, the genetic basis of *comA* was studied in strain *B. subtilis* BSF01. Here, results showed the DNA fragment of 645 bp was obtained by amplifying sequence of designated *comA* gene (Supplementary Figure S1), which encoded a polypeptide of 214 amino acids with a calculated molecular mass of 24.1 kDa and a *pI* of 5.0. On the basis of nucleotide sequence BLAST analysis, *comA* shared 99% of identity to the transcriptional regulator gene *comA* from *B. subtilis* strain 168. Further, the expression of gene *comA* was conducted by inserting the sequence into a pET-SUMO expression vector *E. coli* BL21 (DE3), and the recombinant pET-SUMO-*comA* protein was purified, weighted at approximately 40 kDa with good solubility (Figure 1A). According to BSA calibration (Figure 1B), the concentration of purified ComA recombinant was at 2.0 g mL^–1^. Thanks to the essential role of ComA as response regulator of QS signals in *B. subtilis*, the genetic and transcriptional basis of *comA* in strain BSF01 provided important tools for the subsequent exploration of *comA*-related ecological functions such as xenobiotic biodegradation.

### Characterization of Pyrethroid-Hydrolyzing Carboxylesterase CesB

**FIGURE 2:**
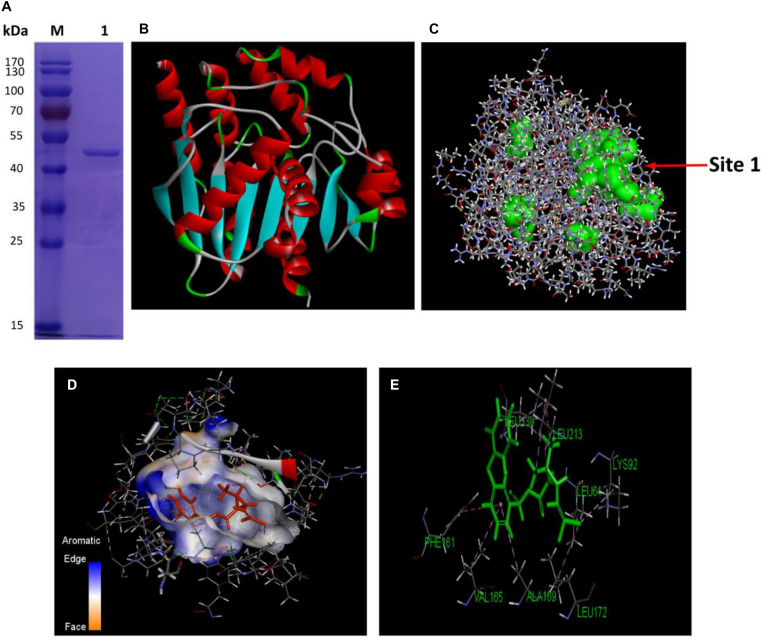
Characterization of CesB. **(A)** The purification of the recombinant CesB protein (M, protein marker; lane 1, purified recombinant); **(B)** 3D model of CesB; **(C)** predicted binding (the arrow indicates binding site 1); **(D)** molecular docking of CesB with β-cypermethrin; and **(E)** key amino acid residues for docking.

Hydrolysis driven by various esterases has been one of the major bioprocesses in microbial degradation of pyrethroids (Bhatt et al., 2020). As a diverse group of hydrolytic enzymes, the carboxylesterases exhibit attractive potential as biocatalysts for biotechnological applications due to their high stability, activity and specificity. In order to find out how the cellular QS network regulated the metabolic functions among bacteria from a transcriptional point of view, the genetic basis and properties of an important esterase, the carboxylesterase CesB encoded by *cesB* gene (also named as *ybfK*) were unveiled in *B. subtilis* BSF01. Firstly, the DNA fragment comprised a single complete ORF of 891 bp of designated *cesB* gene was obtained (Supplementary Figure S2A), encoding a polypeptide of 296 amino acids with a calculated molecular mass of 33.1 kDa and a *pI* of 5.5. According to the nucleotide sequence BLAST analysis, *cesB* shared high identity (98%) to its carboxylesterase gene from *B. subtilis* 168, the carboxylesterase NP (98% identity) from *B. subtilis* BEST7613 and the carboxylesterase YbfK (99% identity) from *B. subtilis* SG6. The multiple alignment comparison among several pyrethroid-hydrolysis enzymes (including Esterase, ACJ07038; PytH, ACM79141; and PytZ, AEY11370) elucidated that CesB protein belonged to the hydrolase family VI with a characteristic α/β hydrolase fold. Besides, the conserved pentapeptide sequence motif Gly–X_1_–Ser–X_2_–Gly (Rozeboom et al., 2014), which was Gly–Phe–Ser–Leu–Gly in this case (Supplementary Figure S2B), is regarded as the typical “nucleophilic elbow” in α/β-hydrolases. Secondly, the recombinant protein CesB was successfully expressed in a pET32a (+) expression vector by inserting *cesB* gene, and the total molecular mass of purified recombinant CesB was determined at approximately 50 kDa with His-, Trx-, and S-tag (Figure 2A). The recombinant protein was soluble and its concentration was determined at 210.8 μg mL^–1^ by the previous BSA calibration.

Further, a reliable three-dimensional (3D) homology model of carboxylesterase CesB was constructed by using CesB from *B. subtilis* (PDB code: 4CCY) which shared the highest similarity (92.9%) with CesB from strain BSF01as a template (Figure 2B). By running the Form Receptor Cavities program, four possible active sites with obvious pocket structures were more likely to bind β-cypermethrin to CesB, among which binding site 1 was located in the largest hydrophobic cavity, showing the highest possibility being the active center for molecular docking (Figure 2C). Indeed, the active site of CesB was located at the interface between the β-cypermethrin and the binding complex in a distance of 4 Å. Based on the results from CDOCKER, the optimal conformation of CesB was determined with a deep, wide and irregular ligand-binding pocket that benefits the β-cypermethrin ligand to enter and bind firmly (Figure 2D). Moreover, the molecular docking revealed several hydrophobic amino acid residues (such as leucine and phenylalanine) around the binding site, constituting the active hydrophobic entrance (Figure 2E). In general, small organic molecules can bind to active proteins by various strategies, such as hydrophobic effect, hydrogen bond and van der Waals force, etc. Since no hydrogen bond was discovered linking the β-cypermethrin ligand to carboxylesterase CesB, they more likely interacted via van der Waals and other electrostatic forces.

**FIGURE 3:**
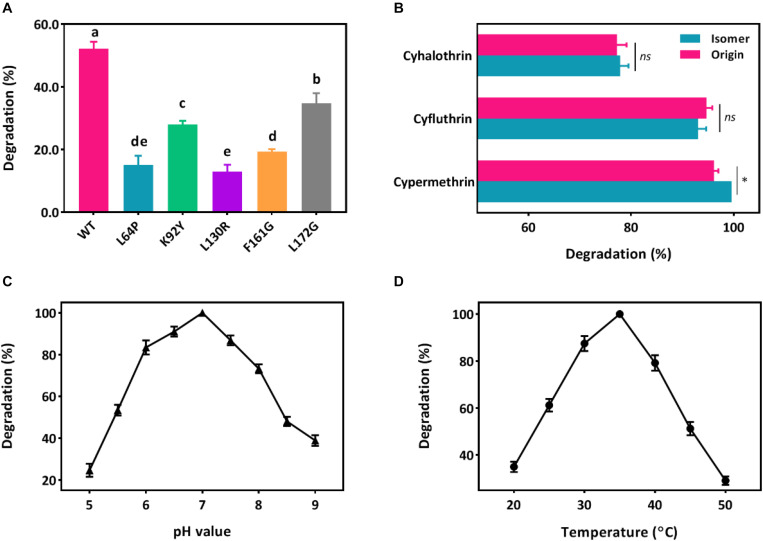
Degradation of pyrethroids by CesB. **(A)** Degradation of β*-*cypermethrin by CesB mutants; **(B)** degradation of cypermethrin, cyfluthrin, cyhalothrin, and their isomers (asterisk presents significant difference within the group, *p* < 0.05); **C**, the effect of pH; and **D**, the effect of temperature. Different letters indicate significant differences among treatments (*p* < 0.05).

Five key amino acid residues including Leu64, Lys92, Leu130, Phe161, and Leu172 were lined in the entrance for the binding, which may cause low affinity or destabilized conformation due to their high mutation energy (Figure 2E and Supplementary Table S2). These five amino acids were individually mutated and the highest mutation energy was recorded when Leu64, Lys92, Leu130, Phe161, and Leu172 were changed into Pro, Tyr, Arg, Gly, and Gly, respectively (Supplementary Tables S3, S4). Then, site-direct mutation of these five amino acids were further conducted using the recombinant pET-32a (+)-*cesB* as a template, namely L64P, K92Y, L130R, F161G, and L172G, respectively. When comparing the degradation efficiency of mutants to the wild-type CesB, five mutants showed significant decline of degradation abilities (Figure 3A), which suggested that the mutation of these five specific amino acid residues could impair the catalytic degradation of CesB possibly due to the destabilized protein conformation and weak binding toward the applied pyrethroid substrate.

CesB has been characterized as an excellent candidate for biocatalytic applications due to its good enantioselectivity. In fact, researchers found that the chirality of pyrethroid compounds can be a key element to influence their ecological toxicity and environmental behaviors (Birolli et al., 2018; Xiang et al., 2019; Yao et al., 2019), which may contribute to broadening its role in pyrethroid biodegradation (Bhatt et al., 2020). As a result, the catalytic ability of CesB from strain BSF01 was evaluated under multiple conditions. First, CesB was able to harness various pyrethroids (including β-cypermethrin, cypermethrin, β-cyfluthrin, cyfluthrin, *λ*-cyhalothrin, and cyhalothrin) as substrates for enzymatic hydrolysis (Figure 3B), suggesting CesB could degrade pyrethroids from a relatively broad spectrum. Interestingly, the catalytic efficiency of CesB toward tested compounds was different but similar between isomers, particularly no specific enantioselectivity was found in the degradation of cyfluthrin, cyhalothrin, and their isomers. This phenomenon is consistent with previous reports that implied the enantioselectivity of CesB toward substrates with chirality in the carboxylic acid part of the esters was relatively low than its homologues (such as CesA) (Oh et al., 2019).

Since *B. subtilis* BSF01 was isolated using β-cypermethrin as a substrate and its carboxylesterase CesB exhibited the highest degradation efficiency toward β*-*cypermethrin, the effects of pH and temperature on the catalytic activity of CesB were investigated using β-cypermethrin as a substrate. As shown in Figure 3C, CesB exhibited β-cypermethrin-degrading activity within a broad pH scope of 5.0–9.0, and more than 70% of its activity remained at pH from 6.0 to 8.0. Meanwhile, the optimal temperature for enzymatic activity was determined from 25 to 45°C (Figure 3D). Accordingly, CesB from strain BSF01 exhibited good stability and catalytic activity toward pyrethroids over a wide range of temperature and pH values, endowing it with promising applications under diverse environment. Taking together, we characterized carboxylesterase CesB from *B. subtilis* BSF01 in terms of its genetic basis, transcriptional features and catalytic function, which established necessary tools to link and unveil the vague interaction between QS regulation and microbial degradation.

#### Synergistic Effect of *comA* and *cesB* in Pyrethriod Degradation

**FIGURE 4:**
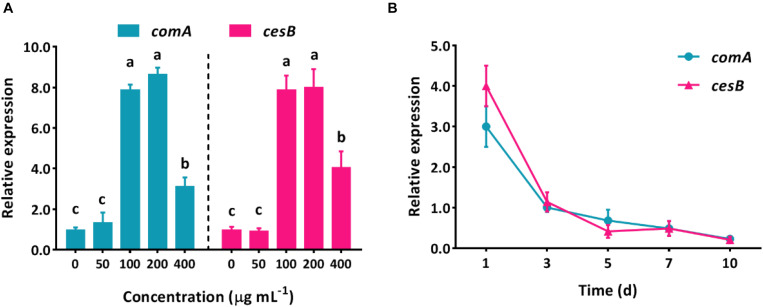
Relative expression levels of QS-related gene *comA* and carboxylesterase gene *cesB* in degrading β-cypermethrin by *B. subtilis* BSF01. **(A)** Expression under β-cypermethrin initially from 0 to 400 μg mL^–1^ (different letters above bars indicate significant differences, *p* < 0.05), and **B**, expression with time.

The spectacular ability that bacteria can harness numerous xenobiotic compounds as nutrient sources has made a myriad of approaches possible for the green elimination of agrochemical contamination without causing side effects. The mechanism of pollutant biodegradation by microorganisms has been deeply studied for decades, but most of them focus on the aspect of an intracellular metabolic process. However, in the natural environment, microorganisms would not perform biological functions individually but collectively, which is highly regulated by QS system. Due to the lack of understanding of how QS system regulates group behaviors to perform biodegradation, the effective utilization of these eco-friendly and functional bacteria (such as *B. subtilis*) would be hindered. Apparently, it is essential to uncover the correlation of intercellular communication system and intracellular metabolic degradation process. Here, in *B. subtilis* BSF01, the relative expression levels of QS-related gene *comA* and its pyrethroid-degrading carboxylesterase gene *cesB* showed similar trends with increasing concentrations of β-cypermethrin (Figure 4A). The expression of both *comA* and *cesB* showed no significant difference treating with β-cypermethrin at 0 and 50 mg L^–1^. However, when exposing bacteria to β-cypermethrin ranging from 100 to 400 mg L^–1^, the expression of *comA* mRNA raised 4.1- to 8.0-fold as against the control and the largest increase of *comA* expression was recorded in the 200 mg L^–1^ treatment. Similarly, by increasing the initial β-cypermethrin to 100–400 mg L^–1^, the carboxylesterase gene *cesB* expressed 3.2–8.7 times more than it in the control, and the expression level also peaked at 200 mg L^–1^ of β-cypermethrin. Actually, these shifts highly suggested that strain *B. subtilis* BSF01 responded to β-cypermethrin by transcriptionally mediating its intracellular catalytic enzyme, in which the ComA-involved QS regulation was closely correlated. Moreover, the expression of two genes were monitored in different intervals, which exhibited similar tendency over temporal scale, unsurprisingly. After 1 day of incubation, the relative expression of *comA* and *cesB* multiplied 3.2- and 4.1-fold as against the control, respectively. As the extension of time, the *comA* and *cesB* expression decreased markedly, showing no difference after the intervals of 3, 5, 7, or 10 days (Figure 4B), which implied that the action of β-cypermethrin degradation could be synchronous with the fluctuation of QS signal levels. Similarly, this kind of intercellular regulation seemed universal in microbial communities since the aerobic bacteria *Methylobacter tundripaludum* was reported to perform environmental methane bio-oxidation by QS system that governing the expression of a secondary metabolite gene cluster (Puri et al., 2017), further suggesting the core role of QS in coordinating ecological functions of potential microorganisms.

**FIGURE 5:**
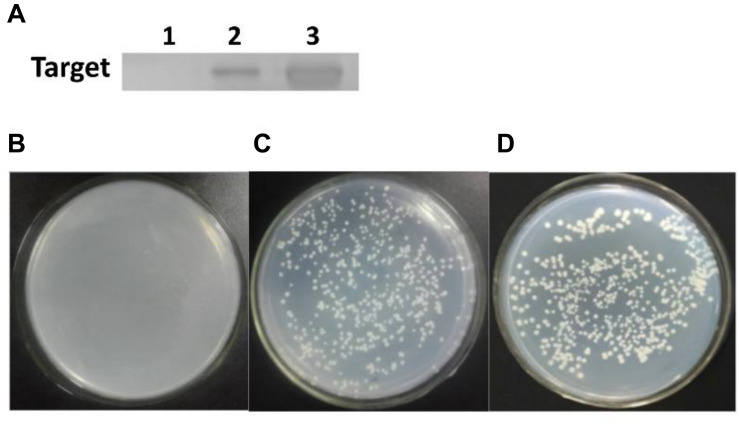
The *cesB*–ComA interaction. **(A)** SDS-PAGE of DNA pull-down. Lane 1, the negative control; lane 2, treatment; lane 3, protein ComA; **(B–D)** growth of recombinant yeasts in Y1H analysis. **(B)** The negative control of plasmids carrying pAbAi-*cesB* and pGADT7; **(C)** treatment of plasmids with pAbAi-*cesB* and pGADT7-*comA*; **(D)** the positive control of plasmids with pAbAi-*cesB* and pGADT7-p53.

But, it remained unclear whether QS signaling exactly triggered the carboxylesterase gene expression and modulate degradation behavior in *B. subtilis* BSF01. Accordingly, the DNA pull-down assay was conducted, and a clear band at 40 kDa was observed in the presence of ComA and the biotinylated *cesB* probe, which verified that ComA specifically bound to the *cesB* fragment (Figure 5A), establishing fundamental basis for the synergistic manipulation of biodegradation function by QS system. Later, the role of ComA as a transcriptional activator was further verified by the Y1H system. In the negative control, the recombinant yeast carrying plasmids of pAbAi-*cesB* and pGADT7 (as a negative control) failed to grow (Figure 5B). Oppositely, the recombinant yeast with plasmids of pAbAi-*cesB* and pGADT7-*comA* and the yeasts of positive control grew normally on the SD/-Leu/AbA medium (Figures 5C,D), which suggested the specific interaction occurred between ComA and *cesB* gene allowing the growth of transformed yeast cells. Indeed, the Y1H analysis evidenced that the ComA protein activated the expression of the reporter gene by binding to the promotor region of *cesB*. Based on these analyses, it elucidated that the ComA-coordinated QS system in *B. subtilis* BSF01 could initiate pyrethroid-degrading function by mediating the expression of carboxylesterase gene *cesB*, providing more mechanistic details about how bacterial social behaviors regulate their ecological role in term of contaminant bioremediation.

In order to maintain and optimize the pyrethroid-degrading potential in bacteria, it is of vital importance to understand the interactions between their intraspecific communication and metabolic functions, which is also consistent with the current demand to cross the border between the well-studied QS mechanisms and practical applications, to solve bottlenecked issues in those functional bacteria-involved technologies. From our results, these interdependent interactions occurred in *B. subtilis* BSF01 supported our original hypothesis that the ComQXPA-involved QS system mediated carboxylesterase CesB at a transcriptional level to perform biodegradation of pyrethroids. Moreover, the action of QS system and enzymatic degradation was synergetic to react to pyrethroid exposure under various conditions, showing the versatility and flexibility of adopting QS-based strategies to enhance functional bacteria-centered approaches for eco-sustainability.

## Conclusion

Due to the impressive catalytic ability of bacteria, biodegradation has long been regarded as one most promising approach to alleviate the ubiquitous contamination stemming from extensive agrochemical use. However, the mechanism of biodegradation as a collective function within bacterial cells remained unclear. In the study, the relation between intercellular QS communication and biodegradation was unveiled using pyrethroid-degrading *B. subtilis* BSF01 as a template. After detailed characterization of the genetic and transcriptional basis of *comA*-involved QS system as well as the pyrethroid-hydrolyzing carboxylesterase CesB, we further evidenced that ComA protein could activate the expression of CesB by binding to the promotor region of *cesB* gene, which subsequently initiated pyrethroid degradation. Taken together, the synergetic regulation of QS system and enzymatic degradation established a novel and fundamental interaction between intraspecific communication and intracellular degradation under xenobiotic exposure, highlighting the tremendous potential of adopting QS-based strategies to enhance functional bacteria-centered approaches for eco-sustainability, which may lead to a new era of research on the basis of intraspecific manipulation and improvement.

## Data Availability Statement

The datasets presented in this study can be found in online repositories. The names of the repository/repositories and accession number(s) can be found in the article/Supplementary Material.

## Author Contributions

YX conducted the experiments. YX and QL analyzed the experimental data. XY helped with figure plotting. YX and JL wrote the original draft. JL revised the draft and coordinated the author team. YX, GZ, and JL provided financial support. All the authors have contributed to the concept and experimental design.

## Conflict of Interest

The authors declare that the research was conducted in the absence of any commercial or financial relationships that could be construed as a potential conflict of interest.

## Footnotes

1 http://www.ncbi.nlm.nih.gov/BLAST

2 http://web.expasy.org/protparam/

3 https://npsa-prabi.ibcp.fr/cgi-bin/npsa_automat.pl?page=npsa_sopm.html

4 https://www.ncbi.nlm.nih.gov/Structure/cdd/cdd.shtml

5 www.bioinformatics.org/primerx/cgi-bin/protein_1.cgi
