# Supplementary material for: Synergistic Degradation of Pyrethroids by the Quorum Sensing-Regulated Carboxylesterase of Bacillus subtilis BSF01
Source: Front Bioeng Biotechnol. 2020 Jul 29;8:889. doi: 10.3389/fbioe.2020.00889 (PMC7403188; doi:10.3389/fbioe.2020.00889)
Supplement: Supplementary file 1 [file Data_Sheet_1.PDF]

Supplementary materials

Synergistic degradation of pyrethroids by the quorum sensing-regulated carboxylesterase of *Bacillus subtilis* BSF01

Ying Xiao [a, b]***, Qiqi Lu*** [a]***, Xin Yi*** [a]***, Guohua Zhong*** [a, c] ****, Jie Liu*** [a] *******

a. Key Laboratory of Integrated Pest Management of Crop in South China, Ministry of Agriculture; Key Laboratory of Natural Pesticide and Chemical Biology, Ministry of Education; South China Agricultural University, Guangzhou 510642, P. R. China

b. Guangdong Research Institute of Petrochemical and Fine Chemical Engineering, Guangzhou 510665, P. R. China

c. Guangdong Laboratory for Lingnan Modern Agricultural Science and Technology, Guangzhou, 510642, P. R. China

*Address correspondence to Guohua Zhong and Jie Liu

E-mail: guohuazhong@scau.edu.cn; jieliu@scau.edu.cn

Tel: +86 020 8528 0308

Table S1 The calibration and spiked recoveries of six pyrethroids

Pyrethroid	Calibration	R^2	Spiked concentration (mg L^{-1})	Average recovery (%)	RSD (%)
β-cypermethrin	y = 54.562x - 60.248	0.9998	1	82.2	3.3
			25	87.5	2.6
			50	94.6	2.4
Cypermethrin	y = 38.082x - 44.181	0.9995	1	87.1	3.3
			25	85.4	0.9
			50	91.1	0.9
β-cyfluthrin	y = 51.984x - 50.939	0.9996	1	81.3	2.0
			25	86.2	2.2
			50	89.9	2.4
Cyfluthrin	y = 53.241x - 38.069	0.9999	1	89.1	5.2
			25	87.7	4.3
			50	88.5	2.9
λ-cyhalothrin	y = 61.130x - 24.732	0.9997	1	91.0	6.7
			25	96.2	5.3
			50	89.6	2.4
Cyhalothrin	y = 59.435x - 28.235	0.9998	1	79.0	2.1
			25	85.7	2.5
			50	89.3	1.2

Table S2 Results of alanine scanning mutagenisis

Mutation	Mutation Energy (kcal/mol)	Effect of mutation
:LEU64>ALA	0.64	Destabilizing
:LEU172>ALA	0.74	Destabilizing
:LYS92>ALA	0.74	Destabilizing
:LEU130>ALA	0.86	Destabilizing
:PHE161>ALA	0.92	Destabilizing

Table S3 Results of saturation mutation

Mutation	Mutation energy (kcal/mol)	Effect of mutation	VDW interaction energy (kcal/mol)	Electrostatic interaction energy (kcal/mol)
:LEU64>PRO	0.77	Destabilizing	1.44	0.08
:LYS92>TYR	1.64	Destabilizing	3.4	0.08
:LEU130>ARG	1.35	Destabilizing	2.57	0.13
:PHE161>GLY	0.97	Destabilizing	1.83	0.02
:LEU172>GLY	0.99	Destabilizing	2.02	-0.02

Table S4 Primer sequences used in this study

Primer function category and name	Primer (5'-3') #	Restriction enzymes
**Gene Cloning**		
comA-F	ATGAAAAAGATACTAGTGATTG	
comA-R	TTAAAGTACACCGTCTGATT	
CesB-F	ATGATACAAGATTCAATGC	
CesB-R	CTATTTTATCCCCCCGCAT	
**Construction plasmids of protein expression**		
comA-eF	CCG**CTCGAG**ATGAAAAAGATACTAGTG	*XhoI* I
comA-eR	CG**GAATTC**TTAAAGTACACCGTCTGAT	*EcoR* I
CesB-eF	CG**GGATCCA**TGATACAAGATTCAATGC	*BamH* I
CesB-eR	CC**AAGCTT**CTATTTTATCCCCCCGCAT	*Hind* III
**Site-directed mutagenesis for CesB**		
T7F	TAATACGACTCACTATAGGG	
T7TER	TGCTAGTTATTGCTCAGCGG	
L64P-F	CTTCACGGGGGCCcTTTCAGCTCTGC	
L64P-R	GCAGAGCTGAAAgGGCCCCCGTGAAG	
K92Y-F	GATATGATAGGAGACtacAATAAAAGTATACC	
K92Y-R	GGTATACTTTTATTgtaGTCTCCTATCATATC	
L130R-F	CTGGCCGGCTTTTCGagaGGCGGGTCCCATATC	
L130R-R	GATATGGGACCCGCCtctCGAAAAGCCGGCCAG	
F161G-F	GCGTTTATTTCAggTCATCCGGATG	
F161G-R	CATCCGGATGAccTGAAATAAACGC	
L172G-F	CTATAAATACGCTGCAGAAggTACAGGGGCAAGTGGAGC	
L172G-R	GCTCCACTTGCCCCTGTAccTTCTGCAGCGTATTTATAG	
**qRT-PCR**		
CesB-RT-F	AGTTTGCCGCGGTTGAAA	
CesB-RT-R	TAAGCGAAGGTGCGTCCT	
comA-RT-F	CAATCAAAACCGCTTCCGTC	
comA-RT-R	GAAATCGCAGATGCCCTTCA	
16s rDNA-RT-F (reference)	TTGCTCCCTGATGTTAGCGGC	
16s rDNA-RT-R (reference)	ACGCATCGTTGCCTTGGTGAG	

Restriction enzymes were underlined; Site-directed mutagenesis was in lower case.

Supplementary figure caption:

Fig. S1 Genetic basis of *comA* in strain BSF01. A, PCR amplification products (M: DNA marker; Lane 1: gene *comA*); and B, Nucleotide sequence and amino acid sequence of *comA* gene

Fig. S2 Genetic and transcriptional basis of *cesB* in strain BSF01. A, PCR amplification products (M: DNA marker; Lane 1: gene *comA*); and B, comparison of multiple sequence alignments among CesB and other homologous enzymes.

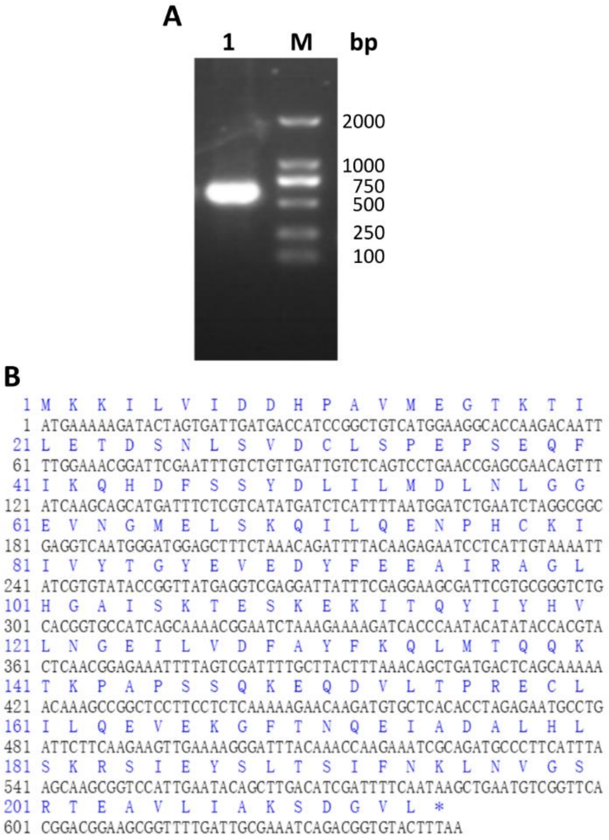

Fig. S1 Genetic basis of *comA* in strain BSF01. A, PCR amplification products (M: DNA marker; Lane 1: gene *comA*); and B, Nucleotide sequence and amino acid sequence of *comA* gene

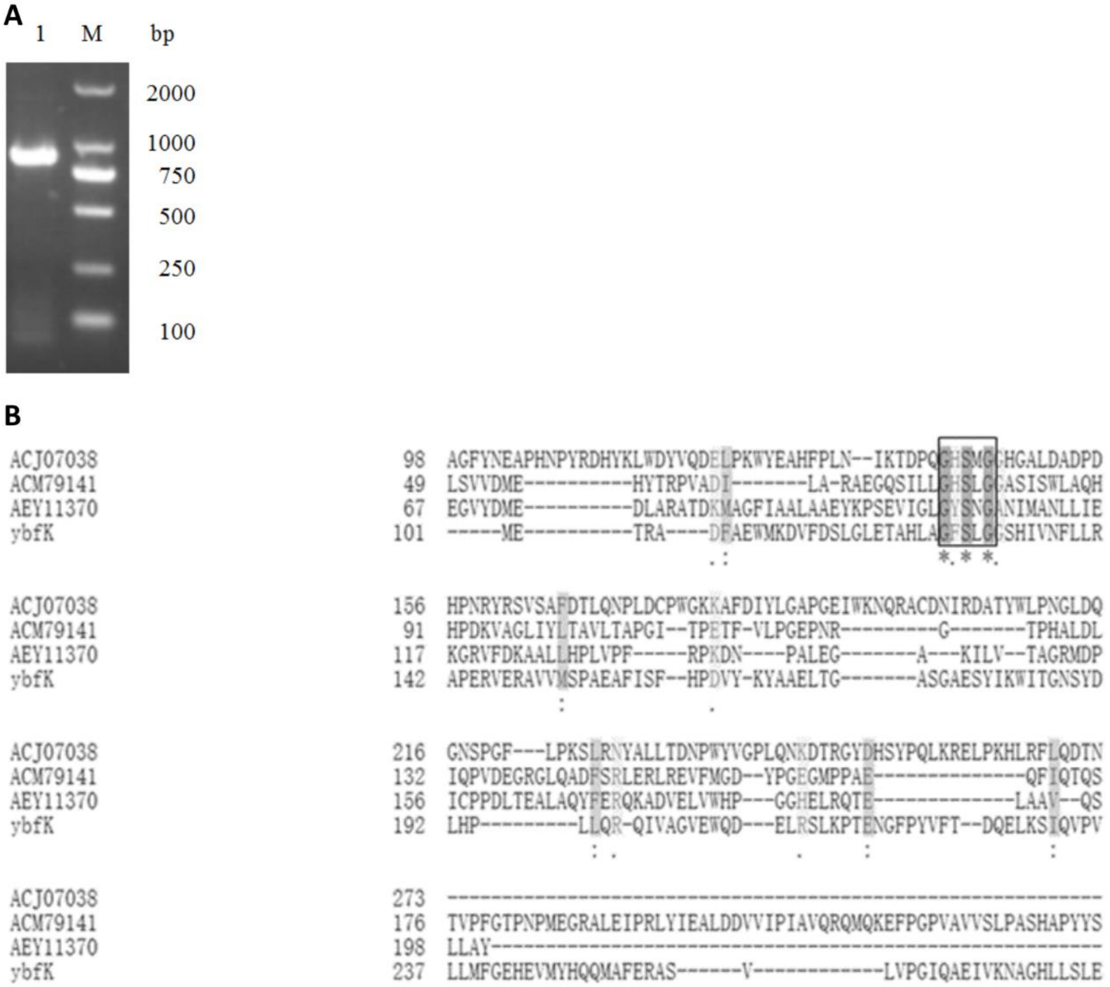

Fig. S2 Genetic and transcriptional basis of *cesB* in strain BSF01. A, PCR amplification products (M: DNA marker; Lane 1: gene *comA*); and B, comparison of multiple sequence alignments among CesB* and other homologous enzymes.

* The alternative name "ybfK" for carboxylesterase CesB was applied during analysis. Its conserved motif Gly-X-Ser-X-Gly was boxed.
